# Dopamine perturbation of gene co-expression networks reveals differential response in schizophrenia for translational machinery

**DOI:** 10.1038/s41398-018-0325-1

**Published:** 2018-12-13

**Authors:** Mark Z. Kos, Jubao Duan, Alan R. Sanders, Lucy Blondell, Eugene I. Drigalenko, Melanie A. Carless, Pablo V. Gejman, Harald H. H. Göring, MGS, P. V. Gejman, A. R. Sanders, J. Duan, D. F. Levinson, J. Shi, N. G. Buccola, B. J. Mowry, R. Freedman, A. Olincy, F. Amin, D. W. Black, J. M. Silverman, W. F. Byerley, C. R. Cloninger, D. M. Svrakic

**Affiliations:** 1 South Texas Diabetes and Obesity Institute, Department of Human Genetics, University of Texas Rio Grande Valley School of Medicine, San Antonio, TX USA; 2 Center for Psychiatric Genetics, NorthShore University HealthSystem, Evanston, IL USA; 3 Department of Psychiatry and Behavioral Neuroscience, University of Chicago, Chicago, IL USA; 4 Department of Genetics, Texas Biomedical Research Institute, San Antonio, TX USA; 5 Molecular Genetics of Schizophrenia (MGS) Collaboration, San Antonio, TX USA; 6 NorthShore University HealthSystem, and University of Chicago, Chicago, IL USA; 7 Stanford University, Stanford, CA USA; 8 National Cancer Institute, Rockville, MD USA; 9 Louisiana State University Health Sciences Center, New Orleans, LA USA; 10 Queensland Centre for Mental Health Research, Brisbane and Queensland Brain Institute, The University of Queensland, St Lucia, Australia; 11 University of Colorado Denver, Denver, CO USA; 12 Atlanta Veterans Affairs Medical Center and Emory University, Atlanta, GA USA; 13 University of Iowa Carver College of Medicine, Iowa City, IA USA; 14 Mount Sinai School of Medicine, New York, NY USA; 15 University of California at San Francisco, San Francisco, CA USA; 16 Washington University, St. Louis, MO USA

## Abstract

The dopaminergic hypothesis of schizophrenia (SZ) postulates that positive symptoms of SZ, in particular psychosis, are due to disturbed neurotransmission via the dopamine (DA) receptor D2 (DRD2). However, DA is a reactive molecule that yields various oxidative species, and thus has important non-receptor-mediated effects, with empirical evidence of cellular toxicity and neurodegeneration. Here we examine non-receptor-mediated effects of DA on gene co-expression networks and its potential role in SZ pathology. Transcriptomic profiles were measured by RNA-seq in B-cell transformed lymphoblastoid cell lines from 514 SZ cases and 690 controls, both before and after exposure to DA ex vivo (100 μM). Gene co-expression modules were identified using Weighted Gene Co-expression Network Analysis for both baseline and DA-stimulated conditions, with each module characterized for biological function and tested for association with SZ status and SNPs from a genome-wide panel. We identified seven co-expression modules under baseline, of which six were preserved in DA-stimulated data. One module shows significantly increased association with SZ after DA perturbation (baseline: *P* = 0.023; DA-stimulated: *P* = 7.8 × 10^-5^; ΔAIC = −10.5) and is highly enriched for genes related to ribosomal proteins and translation (FDR = 4 × 10^−141^), mitochondrial oxidative phosphorylation, and neurodegeneration. SNP association testing revealed tentative QTLs underlying module co-expression, notably at *FASTKD2* (top *P* = 2.8 × 10^−6^), a gene involved in mitochondrial translation. These results substantiate the role of translational machinery in SZ pathogenesis, providing insights into a possible dopaminergic mechanism disrupting mitochondrial function, and demonstrates the utility of disease-relevant functional perturbation in the study of complex genetic etiologies.

## Introduction

Schizophrenia (SZ) is a disabling mental disorder characterized by severe disturbances in thought, behavior, and emotion, including psychotic symptoms and cognitive impairment^1^. Affecting approximately 1% of individuals globally^2^, SZ is heritable and highly polygenic^3^, with a number of neurobiological pathways tentatively implicated in its etiology. The dopaminergic hypothesis is a longstanding SZ model, attributing positive symptoms of the disorder, in particular psychosis, to dysregulation in dopaminergic neurotransmission via the dopamine (DA) receptor D2 (DRD2). This is supported by effects of psychotogenic stimulants (e.g., amphetamines) that activate DA receptors, as in vivo brain imaging studies have shown that amphetamine-induced increases in DA response are correlated with positive symptoms of SZ^4,5^. Moreover, it is well documented that antipsychotic drugs (e.g., chlorpromazine, haloperidol) block DRD2, with clinical response linked to receptor occupancy^6^. Meta-analysis of brain imaging data have shown increased post-synaptic DRD2 density in the striatum of SZ patients, but the relationship is complicated by the absence of significant differences between drug-naïve patients and controls, suggesting that the DRD2 upregulation may be due to antipsychotic treatment^7^. Results from genome-wide association studies (GWAS) do show an association between common variants at the *DRD2* locus and SZ^8^, supporting the contention that D2 receptor variants affect SZ risk.

The action of DA on post-synaptic receptors represents one of the final steps of dopaminergic neurotransmission. A number of studies, however, have identified the most evident dopaminergic abnormality in SZ as being pre-synaptic and likely non-receptor-mediated, related to DA synthesis capacity, baseline synaptic DA levels, and/or DA release. Elevated pre-synaptic striatal DA levels have emerged as a fairly robust feature of SZ^7,9–11^, with increases also observed in the prodromal phase of the disorder that is linked to symptom severity and onset of psychosis^12^. In contrast, recent topographic analyses of extra-striatal brain regions have revealed DA deficits in the dorsolateral prefrontal cortex (DLPFC), with hypofunction associated with the activation of working memory in this region^13^. With current drug treatments primarily acting upon the same mechanism, namely D2/D3 receptor blockade, future research and drug development for SZ is needed to better target pre-synaptic DA abnormalities.

The molecular pathways linking aberrant DA levels to SZ etiology are not well understood. In addition to synaptic transmission, DA is known to have oxidative mechanisms that lead to apoptosis, a process that contributes to DA neuron loss in Parkinson’s disease (PD) and other neurodegenerative disorders^14,15^. Experimental studies provide a potential means of identifying such pathogenic pathways, without confounding due to drug treatment and other factors. Towards this end, we recently investigated non-receptor-mediated effects of DA on cellular gene expression in B-cell transformed lymphoblastoid cell lines (LCLs) and SZ risk. Using a cell perturbation approach ex vivo, we measured transcriptomic profiles by RNA sequencing (RNAseq) before and after exposure to DA in LCLs from SZ cases and controls, revealing differentially expressed genes enriched for brain expression and for functions related to immunity and apoptosis^16^. In this paper, we expand upon this work by examining differences in co-expression patterns (i.e., joint changes in gene behavior) due to DA stimulation. Such network-based approaches offer a means of clustering correlations in the transcriptome that tend to be biologically meaningful and can reveal insights into the larger genetic architecture of complex disorders. Using weighted gene co-expression network analysis, we successfully identified a co-expression module present at both baseline and in the DA-stimulated data, and whose association with SZ varies as a function of DA, with heightened disease correlation upon DA perturbation. This module is highly enriched for ribosomal proteins, as well as for genes implicated in neurodegenerative disorders, providing potential insights into non-receptor-mediated effects of DA in SZ pathogenesis.

## Materials and methods

### Samples

The RNAseq sample consists of 514 SZ cases and 690 controls after quality control (QC) processing (total sample = 1204), as previously described^16^. The subjects are of European ancestry and represent a subset of the Molecular Genetics of Schizophrenia (MGS) collection selected for GWAS and analyses of CNVs and transcriptomics^16–20^. There are 639 males (263 cases and 376 controls) and 565 females (251 cases and 314 controls) in this sample, with study enrollment ages ranging from 15 to 84 years, with detailed phenotypic data available^17,21^. Cases are severely affected on average, with most (~98%) exhibiting positive, psychotic symptoms (i.e., delusions, hallucinations). The NorthShore University HealthSystem Institutional Review Board approved this study, with informed consent obtained from all subjects.

### Cell culture and DA perturbation

LCLs for the study sample were obtained from the Rutgers University Cell and DNA Repository^22^, for which we measured EBV load, viable cell count, and ATP level at cell harvest, all known to have an effect on gene expression^23^. For the design of the DA perturbation model, different DA concentrations were tested in a pilot study of four LCLs from control subjects^16^. At 100 μM, significant changes were observed for gene expression throughout the genome, affecting approximately 13% of genes, with only limited effects on LCL growth (reduction by ~20%), and thus 100 μM was selected for DA perturbation of the larger study sample. Cells were grown in independent wells in the presence or absence of DA, with DA exposure lasting 24 h. For comparison, in vivo DA concentrations in the human striatum, a brain region associated with dopaminergic abnormalities in SZ, is highly varied, with DA levels ranging from nM to μM concentrations, with significant differences between tonic DA release into extra-synaptic spaces (i.e., background activity) and the more transient and intense phasic activation that occurs in the synapses, with interactions between the two mechanisms [Grace, 1991]. Our ex vivo model represents a steady-state of DA exposure, albeit an extreme one, with an intended focus on pre-synaptic and non-receptor-mediated DA effects.

### RNAseq and data processing

RNA sequencing was performed for baseline and DA-stimulated samples at the University of Minnesota Genomics Center on an Illumina HiSeq2000 at approximately 10 million reads per sample, with RNA quality scores indicative of high quality. Resulting RNAseq data were processed as previously described^16,20,24^. Alignment of 50-bp single reads to the Gencode v. 20 (GRCh38) human genome reference assembly was achieved with Tophat v. 2.0.5. Gene expression levels were calculated as reads per kilobase (kb) of transcript, per million mapped reads (RPKM) for the exon model of the longest transcript of a gene, with each quantile-normalized to account for batch biases and log2 transformed for variation stabilization. QC of gene RPKM levels, as previously described in detail^16^, involved several steps, including analysis of technical and biological replicates (i.e., same RNA and independent cell cultures of same LCL sample), consistency of sex chromosome gene expressions versus reported sex, comparison of RNAseq-called genotypes with previous GWAS SNP genotypes^17,18^, comparison of sample completion rate between cases and controls for each gene at baseline or DA-stimulation conditions, and identification of expression outliers based on PCA. QC processing yielded gene expression data for 21,043 genes for both baseline and DA-stimulated conditions. For downstream analyses, log2 transformed RPKM expression levels were residualized for the following relevant covariates: sex, age, cell counts and ATP levels at cell harvest under the two respective conditions, genotypic ancestry PCs 1–5, Epstein-Barr virus load, and sequencing batch. The variance in gene expression explained by these biological and technical confounds are presented in Supplementary Figures 1 and 2, with non-significant differences observed in the overall variance estimates for the baseline and DA-stimulated data sets (s^2^_baseline_ = 4.20; s^2^_DA_ = 4.18; *P* = 0.48).

### Weighted gene co-expression network analysis (WGCNA)

To identify correlation patterns in the expression data for baseline and DA-stimulated conditions, WGCNA was performed using the R package of the same name (WGCNA v. 3.3.3)^25^. The WGCNA pipeline is as follows: (1) Using the command “pickSoftThreshold”, we assessed whether gene expression data have “scale-free topology” (i.e., frequency distribution of *k*, which is the summation of pairwise correlation coefficients for each gene, follows a power law) and identified values of the exponential parameter *β* for achieving it. A transformation such as this down-weights weaker correlations between genes, resulting in more cohesive co-expression networks that are centered on and stabilized by highly connected “hub genes”, which the authors of this approach argue are robust to random changes in connection patterns and more closely resemble true biology^26^. (2) Adjacency matrices were computed for each data set (i.e., baseline and DA-stimulation), representing pairwise correlation coefficients (i.e., Pearson’s *r*) transformed by the aforementioned *β* to ensure a scale-free correlation structure. We employed the unsigned method, in which *absolute* values of the coefficients were transformed, thus avoiding ablation of any strongly *negative* co-expression relationships (note: we also performed WGCNA based on signed adjacency matrices, in order to investigate the robustness of our identified modules and their associations with SZ). (3) From the adjacency matrices, topological overlap matrices (TOMs) were computed, representing the “interconnectedness” between pairs of genes, both directly, as well as indirectly, with connection strengths mediated by shared gene neighbors that are one-step away, which reportedly achieves more cohesive and biologically meaningful modules than ones determined solely from direct correlations^27^. These values were then used to calculate a dissimilarity distance measure, DistTOM, equating to 1—TOM. (4) We constructed dendrograms for the 21,043 genes with available gene expression data based on hierarchical clustering of DistTOM scores. This was achieved using the well-established UPGMA method via the R command “hclust”. Modules of co-expressed genes were then determined from branches in the resulting dendrograms using the command “cutreeDynamic”, which performs adaptive branch pruning based on various criteria, including a minimum cluster size of 50 genes and a conservative branch cut height of 0.99. The Partitioning Around Medoids option, which is a greedy algorithm for identifying outlying genes for module inclusion, was not utilized in order to maintain cohesive modules within the dendrograms. With these particular settings, modules of highly correlated genes will be preferentially detected, with the larger number of genes exhibiting weaker network connectivities left unassigned, including potential risk genes. (5) Module eigengenes (ME) were computed for each subject, which simply represent the first principal component of expression levels of genes assigned to a particular module^28^. ME scores were then used to calculate pairwise correlations between modules, as well as with SZ status, which in turn were used to generate dissimilarity distance matrices (DistME) by subtracting the coefficients from one and construct dendrograms via UPGMA. A distance threshold of 0.25 was used to identify closely related modules, which were then merged, necessitating the recalculation of MEs for final module assignments.

WGCNA results were visualized through a combination of dendrograms and heatmaps (R commands “TOMplot” and “plotEigengeneNetworks”), as well as network topologies comprised of gene nodes and edges (i.e., gene-gene connection strengths from the adjacency matrices; threshold = 0.01) that were constructed in Cytoscape v. 3.6.0^29^. Significance of gene overlap between modules across the two conditions was determined through pairwise hypergeometric testing of 2 × 2 contingency tables (i.e., Fisher’s exact test). Modules were originally assigned random color names, which were subsequently changed such that modules with significant overlap have matching color names, in an effort to simplify for the reader the downstream analyses that compare WGCNA results across the two conditions.

### Association between WGCNA modules and SZ

The relationships of the baseline modules to SZ status (i.e., case or control) were examined through logistic regression analysis of the respective ME scores in R v. 3.3.2 (note: no other covariates were included since gene expression data were residualized; see above), as well as for recomputed MEs based on the DA-stimulated data for the same set of module genes. Association results for the two sets of MEs were compared, with goodness-of-fit assessed by differences in Akaike Information Criteria (ΔAIC) for the DA-stimulated and baseline models (note: due to multicollinearity, multiple regression models that include both MEs were not examined). Moreover, we calculated the proportion of genes in each module that have been linked to SZ risk in the large GWAS conducted by the SZ Working Group of the Psychiatric Genomics Consortium (PGC)^8^. The 108 SNPs and indels that displayed genome-wide significance in the PGC study were assigned to 1308 genes, including non-protein coding RNAs, using a 250 K bp window around the loci in the GRCh38 reference assembly, with the significance of the enrichments determined via permutations (10,000×) weighted by total gene connectivity scores (*k*) from the adjacency matrices.

### Preservation of WGCNA modules

How well the baseline modules were preserved under DA-stimulation (and vice versa) was assessed in the WGCNA program through various statistics related to module density and connectivity in the two data sets^30^, including correlations of: intramodular connectivity (*kIM*), which is the summation of adjacency matrix values, or connection strengths, of a module gene with other module genes; and eigengene-based connectivity (*kME*), representing the correlation of gene expression profiles with MEs of a given module. To summarize the different preservation metrics, a composite statistic based on median rank was computed for each module. The *kIM* scores were also used to screen for so-called “hub genes”, highly connected nodes that may represent key drivers in the co-expression modules.

### Gene set enrichment analysis

To identify classes of genes or biological features that are over-represented in our WGCNA modules, we performed enrichment analyses for gene lists defined by GO-terms (molecular functions and biological processes) and KEGG pathways using the online tool DAVID v. 6.8^31^, relative to the total set of genes comprising our expression data. Significance of gene enrichment was evaluated using a modified, and more conservative, Fisher’s Exact *P*-value, as well as false discovery rate (FDR). In addition, we employed the functional annotation clustering algorithm available in DAVID, which accounts for the redundant nature of gene annotations, and reports groups of related annotation terms from across various bioinformatics databases, yielding enrichment scores representing the geometric means of Fisher’s Exact *P*-values for individual gene sets tested in the primary analyses.

### Genome-wide association testing

We screened the genome for quantitative trait loci (QTL) influencing co-expression patterns of select WGCNA modules. Genotype association testing was performed on ME scores in PLINK v. 1.07 via linear regression^32^, which included the first five principal components as covariates to control for potential population substructure. The genotype data were generated on the Affymetrix 6.0 array, with 671,422 SNPs passing QC filtering, as previously described^17^. In addition, we tested for SNP*SZ interaction in the linear model in an effort to identify QTL effects that are modified by case-control status (i.e., comparing the SNP regression coefficients of the two groups).

## Results

### WGCNA modules

While we have previously identified a large number of genes whose expression level response to DA perturbation varies between LCLs from SZ cases compared to ones from unaffected individuals^16^, the interpretation of these transcriptome-wide results are complex. We therefore employed an alternative analytical approach, one that focuses on gene co-expression networks—and thus presumably biology—to potentially yield additional insights. The first step in WGCNA was to evaluate the scale-free topology of our gene expression data sets. This is characterized by a correlation structure in the data that is comprised of highly connected hub genes, with relatively weaker pairwise correlations outside the hubs. Neither the baseline nor the DA-stimulated data are scale-free according to the WGCNA standard, which is the norm for gene expression studies. Thus, the baseline and DA data sets needed to be transformed for the adjacency matrices by down-weighting weaker correlations, with the *β* parameter set to 8 and 10, respectively, to achieve an *r*^2^ ≥ 0.9 with the scale-free model. Given our interest in the *changes* in gene expression in response to DA-stimulation (and whether it varies between SZ cases and controls), we also evaluated differential expression levels between the two conditions (i.e., response to DA perturbation), which were the target phenotype of a previous paper reporting on transcriptomic signatures of SZ by our research group^16^. However, the response data are not scale-free, and we failed to achieve robust fits to the scale-free model when transformed under a series of *β* powers (ranging from 1 to 20) for the total sample, as well as for SZ cases and controls separately. The authors of the WGCNA approach recommend against using such differential data, as it alters gene-gene co-expression structure and leads to single, highly correlated networks, invalidating the scale-free assumption. Thus, we performed WGCNA on the baseline and DA-stimulated data sets *separately*, and then compared the resulting modules.

**Fig. 1:**
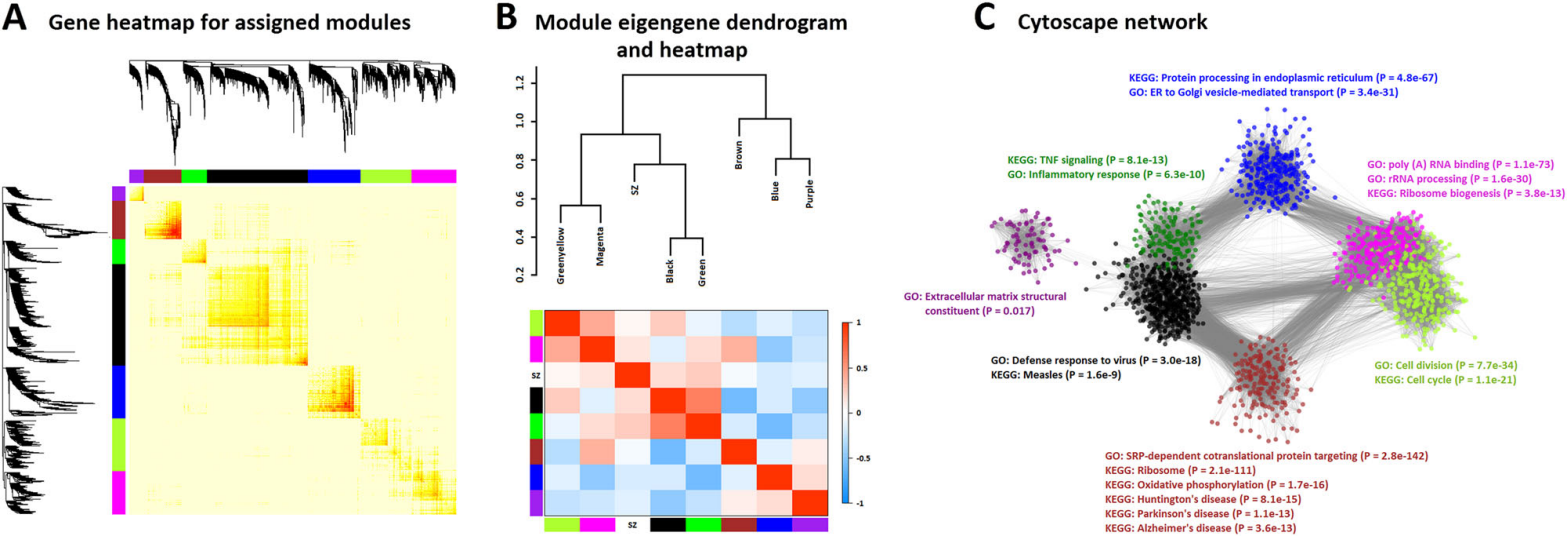
**a** Heatmap of pairwise TOM scores, aligned with the relevant dendrogram branches, of genes assigned to the seven baseline modules. **b** Correlation heatmap and dendrogram of eigengene profiles for baseline modules (including SZ status). **c** Two-dimensional network of gene-gene connection strengths (adjacency matrix values; minimum of 0.01) created in Cytoscape v. 3.6.0. Node colors correspond to baseline modules to which the genes were assigned. Top results from gene enrichment analyses of GO-terms and KEGG pathways are also shown, with Fisher’s Exact *P*-values in parentheses

For the baseline data, 13 co-expression modules were initially identified, each randomly assigned to a color (Supplementary Figure 3), which were reduced to seven after merging modules with similar expression profiles (DistME < 0.25). The sizes of the seven modules range from 74 to 521 genes, with mean intramodular *k* values ranging from 1.17 to 8.61 (overall mean of 5.44), indicating that the identified modules comprise of highly intercorrelated genes. Most of the genes in the dataset are unassigned (*n* = 19,366), labeled as “grey”. As is evident from the deep branching in the dendrogram and the corresponding red highlights in the heatmap in Fig. 1a, the brown and blue modules exhibit the strongest gene-gene interconnectedness based on TOM dissimilarity distances. When comparing modules based on their eigengene profiles (Fig. 1b), the black and green modules show the strongest connections between one another (DistME = 0.39), sharing a clade with the green-yellow and magenta modules. To better visualize the gene co-expressions beyond what are represented in these binary-tree hierarchies, we constructed a two-dimensional network topology in Cytoscape v. 3.6.0 (Fig. 1c). With a minimum threshold of 0.01 for gene-gene connection strength, the purple module emerges as an outlier, with only a small number of network edges paired with black nodes. Overall, the network shows where connections are most concentrated (e.g., between the brown and black modules) and where they are not (e.g., between the brown and blue modules).

**Fig. 2:**
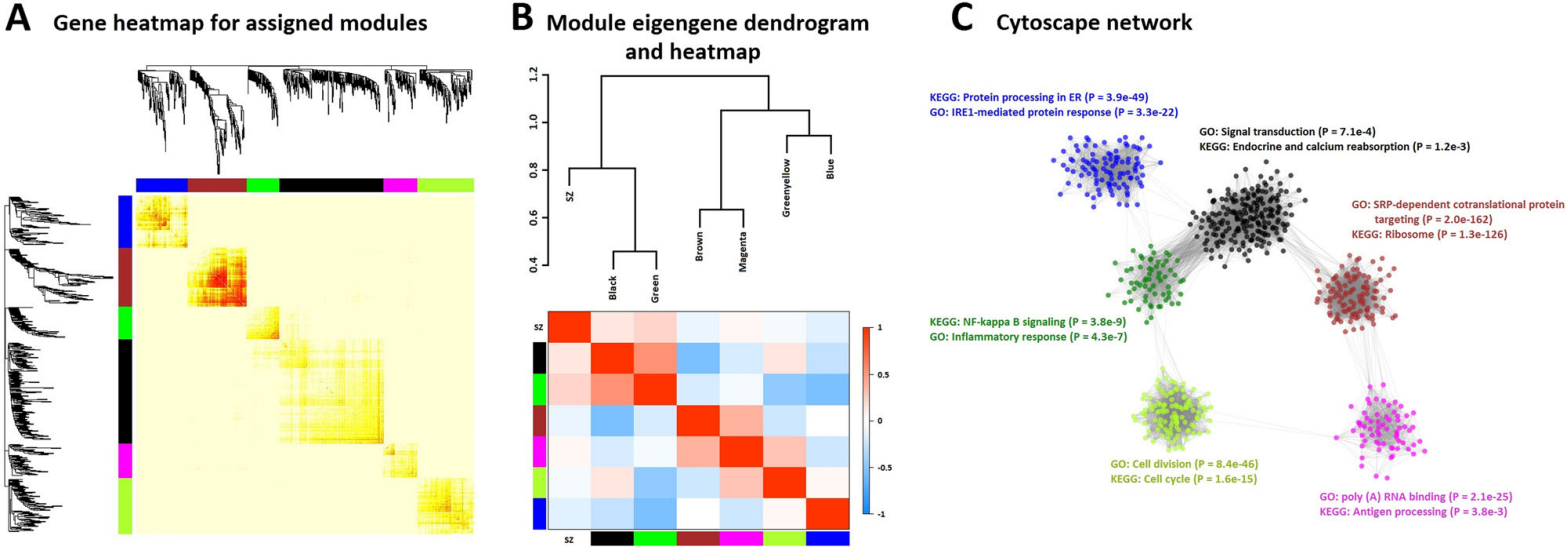
**a** Heatmap of pairwise TOM scores, aligned with the relevant dendrogram branches, of genes assigned to the six DA-stimulated modules. **b** Correlation heatmap and dendrogram of eigengene profiles for DA-stimulated modules (including SZ status). **c** Two-dimensional network of gene-gene connection strengths (adjacency matrix values; minimum of 0.01) created in Cytoscape v. 3.6.0. Node colors correspond to DA-stimulated modules to which the genes were assigned. Top results from gene enrichment analyses of GO-terms and KEGG pathways are also shown, with Fisher’s Exact *P*-values in parentheses

For the DA-stimulated data, seven co-expression modules were initially identified (Supplementary Figure 4), which were reduced to six after merging of two similar modules. The six modules range from 62 to 198 genes. Relative to the baseline results, the number of unassigned genes (*n* = 20,401) increased by 1,035 genes, perhaps indicating transcriptome-wide disruption in co-expression patterns upon DA exposure of the LCLs. To understand how these modules relate to the ones generated in the baseline data, we performed pairwise hypergeometric testing across the two sets, thus identifying modules that show significant overlap in gene membership (Table 1). All six DA-stimulated modules were found to have substantial and exclusive overlap with a baseline module, ranging from 86.9% to all of the genes being shared. Similar to the baseline, the brown DA-stimulated module displays the strongest connectivity in the gene heatmap, harboring the deepest branches in the corresponding dendrogram (Fig. 2a). The black and green modules again were closest in their co-expression levels (Fig. 2b; DistME = 0.46), which is evident in the Cytoscape network (Fig. 2c), with most of the edges positioned between these two modules.

**Table 1:** Overlap between baseline and DA-stimulated WGCNA modules

Module color	No. genes—baseline	No. genes—DA-stimulated	Overlap (%)^a^	*P*-value^b^
Black	521	198	97.5	3.4 × 10^−322^
Blue	270	99	100.0	0^c^
Brown	194	111	99.1	6.2 × 10^−244^
Green	127	62	95.2	6.9 × 10^−138^
Green-Yellow	264	107	86.9	2.1 × 10^−171^
Magenta	227	65	90.8	3.4 × 10^−115^
Purple^d^	74	–	–	–

^a^Percent of genes in the DA module that overlaps with genes in the respective baseline module

^b^*P*-values based on the hypergeometric test, as computed by the R command “overlapTableUsingKME” in the WGCNA package

^c^Hypergeometric *P*-value too small to be estimated

^d^All 74 genes in the purple baseline module were unassigned in the WGCNA results for the DA-stimulated data

Given the significant overlap in the genes comprising the baseline and DA-stimulated modules, it is perhaps not surprising that the gene-gene interconnectedness of the baseline modules is well preserved in the DA-stimulated expression data (Supplementary Table 1), with strong correlations in both *kIM*, ranging from 0.78 to 0.99, and *kME*, ranging from 0.82 to 0.99 (excluding the purple module outlier). Moreover, for three of the six matching modules, the same hub genes are identified based on the top *kIM* scores (Supplementary Table 2): *RPL11* for the brown modules; *HNF1b* for the black modules; and *NFKB2* for the green modules. The median ranks of the various preservation statistics from the WGCNA program indicate that the brown module is the best preserved of the seven baseline modules in the DA-stimulated data, and vice versa.

### Gene set enrichment analyses

To investigate the potential biological relevance of the WGCNA modules, we performed gene set enrichment analyses for GO-terms and KEGG pathways in DAVID (Table 2). For both the baseline and DA-stimulated results, highly significant, replicated enrichments were observed in the pair of matching brown modules: GO-term “SRP-dependent co-translational protein targeting to membrane”, representing 42.3% and 70.6% of the genes, respectively (FDR = 4.0 × 10^−141^ and 2.7 × 10^−161^); and KEGG pathway “Ribosome”, representing 40.7% and 68.8% of the same genes (FDR = 2.3 × 10^−110^ and 1.1 × 10^−125^). This is also reflected in the functional annotation clustering of the entire enrichment results (Supplementary Table 3), as the brown modules produced the highest overall scores in each data set for terms related to ribosomal translation. Some of the other top enrichments that were observed in both matching modules include: KEGG pathway “Protein processing in endoplasmic reticulum” for the blue modules; GO-term “Inflammatory response” for the green modules; KEGG pathway “Cell cycle” and GO-term “Cell division” for the green-yellow modules; and GO-term “Poly(A) RNA binding” for the magenta modules. Among the top enrichment results that are *not* shared, most remain highly enriched in the alternate module but are simply not the top hit (e.g., TNF signaling is the second highest enriched KEGG pathway in the DA-stimulated green module; FDR = 4.0 × 10^−8^). Although DA-stimulation does appear to have broadly weakened the top enrichment signals observed in the baseline modules, some dramatically (e.g., GO-term “Defense response to virus” in the black modules, with the baseline FDR of 5.2 × 10^−17^ becoming a non-significant 0.59), with the exceptions being the GO-term “Cell division” for the green-yellow modules and the top results from the brown modules.

**Table 2:** Top gene set enrichment for WGCNA modules

Module	Top KEGG pathway	FDR	Top GO-term^a^	FDR
	Baseline			
Black	hsa05162: Measles	2.0 × 10^−8^	0051607: Defense response to virus	5.2 × 10^−17^
Blue	hsa04141: Protein processing in ER	5.4 × 10^−66^	0006888: ER to Golgi vesicle transport	5.3 × 10^−30^
Brown	hsa03010: Ribosome^b^	2.3 × 10^−110^	0006614: SRP protein targeting to ER^c^	4.0 × 10^−141^
Green	hsa04668: TNF signaling	9.6 × 10^−12^	0006954: Inflammatory response	9.7 × 10^−9^
Green-Yellow	hsa04110: Cell cycle	1.2 × 10^−20^	0051301: Cell division	1.2 × 10^−32^
Magenta	hsa03008: Ribosome biogenesis	4.3 × 10^−12^	0044822: Poly(A) RNA binding	1.5 × 10^−72^
Purple	hsa04960: Sodium reabsorption	0.63	0005201: Extracellular matrix structure	0.18
	DA-stimulated			
Black	hsa04961: Endocrinal calcium reabsorption	0.014	0007165: Signal transduction	0.011
Blue	hsa04141: Protein processing in ER	3.1 × 10^−48^	0036498: IRE1-mediated protein response	4.4 × 10^−21^
Brown	hsa03010: Ribosome	1.1 × 10^−125^	0006614: SRP protein targeting to ER	2.7 × 10^−161^
Green	hsa04064: NF-kappa B signaling	4.1 × 10^−8^	0006954: Inflammatory response	6.4 × 10^−6^
Green-Yellow	hsa04110: Cell cycle	1.3 × 10^−14^	0051301: Cell division	1.2 × 10^−44^
Magenta	hsa04612: Antigen processing	0.037	0044822: Poly(A) RNA binding	2.6 × 10^−24^

^a^Tested GO-terms related to biological processes and molecular functions in DAVID v. 6.8

^b^Genes from the brown baseline module belonging to this pathway (*n* = 80): MRPL33, MRPL34, MRPS21, RPL10, RPL10A, RPL11, RPL12, RPL13, RPL13A, RPL14, RPL15, RPL17, RPL18, RPL18A, RPL19, RPL21, RPL22, RPL22L1, RPL23, RPL23A, RPL24, RPL26, RPL27, RPL27A, RPL28, RPL29, RPL3, RPL30, RPL31, RPL32, RPL34, RPL35, RPL35A, RPL36, RPL36A, RPL37, RPL37A, RPL38, RPL39, RPL4, RPL41, RPL5, RPL6, RPL7, RPL7A, RPL8, RPL9, RPLP0, RPLP1, RPLP2, RPS10, RPS11, RPS12, RPS13, RPS14, RPS15, RPS15A, RPS16, RPS18, RPS19, RPS2, RPS20, RPS21, RPS23, RPS24, RPS25, RPS27, RPS27A, RPS28, RPS29, RPS3, RPS3A, RPS4X, RPS5, RPS6, RPS7, RPS8, RPS9, RPSA, and UBA52

^c^Genes from the brown baseline module belonging to this GO-term (*n* = 77): RPL10, RPL10A, RPL11, RPL12, RPL13, RPL13A, RPL14, RPL15, RPL17, RPL18, RPL18A, RPL19, RPL21, RPL22, RPL23, RPL23A, RPL24, RPL26, RPL27, RPL27A, RPL28, RPL29, RPL3, RPL30, RPL31, RPL32, RPL34, RPL35, RPL35A, RPL36, RPL36A, RPL37, RPL37A, RPL38, RPL39, RPL4, RPL41, RPL5, RPL6, RPL7, RPL7A, RPL8, RPL9, RPLP0, RPLP1, RPLP2, RPS10, RPS11, RPS12, RPS13, RPS14, RPS15, RPS15A, RPS16, RPS18, RPS19, RPS2, RPS20, RPS21, RPS23, RPS24, RPS25, RPS27, RPS27A, RPS28, RPS29, RPS3, RPS3A, RPS4X, RPS5, RPS6, RPS7, RPS8, RPS9, RPSA, SRP14, and UBA52

### Associations with SZ

The gene network modules were initially identified and subsequently characterized using the entire gene expression data sets, without taking SZ status into account. Thus, to determine whether the co-expression levels of a module differ between cases and controls, ME scores for the baseline modules (proportion of the variation explained ranging from 0.48 to 0.57) were tested for association with SZ (Table 3). With the exception of the green-yellow (*P* = 0.11) and brown modules (*P* = 0.023), the gene co-expressions of the baseline modules show highly significant associations with SZ (*P* < 10^−5^), with the green module, which is enriched for genes related to inflammatory response (see above), having the most significant result (*P* < 2.0 × 10^−16^). This is in general agreement with our previous report which found a vast number of genes to be differentially expressed between cases and controls at baseline^16^. To investigate the impact of DA-stimulation on these findings, we recomputed the MEs using the DA-stimulated expression data for the exact same sets of module genes (accounting for 0.42–0.52 of the variation), and then retested the associations. In other words, we compared our two conditions by generating respective pairs of co-expression profiles for each baseline module and thus fixed for gene membership. For six of the modules, less significant associations were observed, reflected by decreases in the estimated regression coefficients. The lone exception is the brown module (enriched for translational machinery), for which the *P*-value decreased from 0.023 to 7.8 × 10^−5^, corresponding to an increase in absolute effect size from −1.12 ± 0.96 to −1.95 ± 0.96 (*Z* = 1.19; *P* = 0.11). This module is also the only one exhibiting greater goodness-of-fit between SZ and its DA-stimulated MEs (ΔAIC = −10.5), representing approximately 190-fold increase in model likelihood^33^. As one would expect, similar association results were observed for MEs from the matching DA-stimulated modules (Supplementary Table 4). Notably, 4.1% of the genes in the brown module are located within 250 K bp of the 108 SZ risk loci reported by the PGC^8^, which is the highest percentage among the seven modules, and represents near significant enrichment relative to the 2.5% observed for unassigned genes in the baseline data set (empirical *P* = 0.079).

**Table 3:** Associations between baseline co-expression modules and SZ

			DA-stimulated data^a^			
Baseline module	Beta (SE)	*P*-value	Beta (SE)	*P*-value	ΔAIC^b^	% PGC loci^c^
Black	3.30 (0.49)	1.7 × 10^−11^	3.00 (0.49)	9.8 × 10^−10^	7.9	2.7
Blue	−3.07 (0.49)	4.1 × 10^−10^	−2.63 (0.49)	8.7 × 10^−8^	10.4	1.1
Brown	−1.12 (0.49)	0.023	−1.95 (0.49)	7.8 × 10^−5^	−10.5	4.1
Green	4.46 (0.48)	<2.0 × 10^−16^	3.95 (0.49)	6.0 × 10^−16^	18.4	1.6
Green-Yellow	0.78 (0.49)	0.11	0.092 (0.49)	0.85	2.5	2.7
Magenta	2.29 (0.49)	3.4 × 10^−6^	0.98 (0.49)	0.048	17.7	4.0
Purple	−2.55 (0.49)	2.3 × 10^−7^	−2.16 (0.49)	1.2 × 10^−5^	7.7	0
Grey (Unassigned)	3.24 (0.49)	4.3 × 10^−11^	2.44 (0.49)	7.2 × 10^−7^	18.9	2.5

^a^For the baseline WGCNA modules, eigengenes were recalculated based on the DA-stimulated gene expression data, which were then tested for association with SZ status

^b^Difference in Akaike Information Criteria (AIC) values for the DA-stimulated and baseline regression models

^c^Percentage of SZ risk genes in a given module. This is based on the findings of the PGC GWAS on SZ, in which 108 SNPs and indels were identified as genome-wide significant, which were assigned to genes and ncRNAs using a 250 K bp window around the loci. The percentage of unassigned genes that are PGC risk genes (as defined above) is 2.5%

### Signed WGCNA networks

The modules reported here are based on unsigned networks, which incorporate negative correlations between genes. To determine how this potentially impacted our network construction (i.e., TOM calculations) and association test results, we re-ran WGCNA using a *signed* adjacency matrix for the baseline data. All seven of the original baseline modules were found to have matching modules from the signed network, with highly significant overlaps in gene memberships (*P* < 1.9 × 10^−163^), and each corresponding to the same top enrichments in GO-terms and KEGG pathways (e.g., brown module: SRP-dependent co-translational protein targeting, FDR = 3.6 × 10^−117^; and ribosome, FDR = 5.5 × 10^−105^), except for the purple module. Moreover, the matching green module from the signed network is again the most strongly associated with SZ (*P* < 2.0 × 10^−16^), and similarly the brown module is the only one exhibiting a marked increase in association upon DA-stimulation, producing an identical ΔAIC of -10.5, which is again a stark outlier among all the other identified modules (ΔAIC ranges from −0.4 to 21). All of this underscores the robustness of our key findings.

### QTLs for the brown module

Given that the brown module is the only module that exhibits stronger association with SZ upon DA-stimulation, with preserved connectivity patterns and gene enrichment profile from the baseline condition, we scanned its co-expression levels for QTLs, representing genetic “drivers” of regulatory systems that may underlie the architecture of transcriptional patterns (e.g., transcription factors, non-coding RNAs (ncRNAs), feedback mechanisms), by testing its ME scores for association with genome-wide SNP data (*n* = 671,423) available for the study sample. The top-5 association results for the brown module under DA-stimulation are presented in Table 4, with the MEs accounting for 64% of the variation. None achieved genome-wide significance (FDR = 0.35; genomic inflation factor *λ* = 1.0), with the top SNP, rs6504934 (*P* = 2.7 × 10^−6^), located upstream (~18 kb) of a long non-coding RNA (lncRNA), RN7SKP14. For the other four SNPs listed in the table, all are in close proximity and exhibit strong LD (pairwise *r*^2^ > 0.87), located within or near *FASTKD2*. This gene encodes a pro-apoptotic protein that is required for mitochondrial ribosome assembly^34,35^, and has been associated with memory performance and hippocampal structure^36^.

**Table 4:** Top-5 genome-wide SNP associations with brown module eigengenes for dopamine-stimulated data

SNP	Chrom.	Position (bp)^a^	MA^b^	Gene/ncRNA (Distance)	Beta (SE)	*P* ^c^
rs6504934	17	54,752,745	A	*RN7SKP14* (18 kb)	0.0057 (0.0012)	2.7 × 10^−6^
rs17280449	2	206,821,737	T	*FASTKD2*	−0.0090 (0.0019)	2.8 × 10^−6^
rs6726245	2	206,814,503	A	*FASTKD2*	−0.0090 (0.0019)	2.8 × 10^−6^
rs16838820	2	206,755,606	C	*MDH1B*; *FASTKD2* (10 kb)	−0.0093 (0.0020)	3.1 × 10^−6^
rs6435351	2	206,794,290	C	*FASTKD2*	−0.0093 (0.0020)	3.3 × 10^−6^

^a^Based on human reference assembly GRCh38.p7

^b^Minor allele

^c^For the *P*-values presented here, FDR (Benjamini-Hochberg method) = 0.35

We also tested for interaction between SNP genotypes and case-control status on co-expression scores in order to assess whether QTL effects vary as a function of SZ under DA-stimulation. The top-5 SNP*SZ interaction effects are given in Table 5 (note: results for the baseline data are available in Supplementary Table 5). Again, none achieved genome-wide significance (FDR = 0.18; *λ* = 0.99). The top SNP, rs10497316 (*P* = 3.8 × 10^−7^), is located in the gene *XIRP2*, which encodes an actin-binding protein, and does not show evidence of association with SZ in the PGC GWAS results, although strong enrichment of rare loss-of-function (LoF) mutations in *XIRP2* were observed among SZ cases in a recent whole-exome sequence study^37^. For the other SNPs listed in the table, they are located either within or closest to lncRNA genes, with three in near-perfect LD (pairwise *r*^2^ > 0.99), and all four showing nominal evidence of association with SZ in the PGC data (*P*-values ranging from 0.050 to 0.0043).

**Table 5:** Top-5 Genome-wide SNP × SZ interactions for brown module eigengenes for dopamine-stimulated data

SNP	Chrom.	Position (bp)^a^	MA^b^	Gene/ncRNA (Distance)	Beta (SE)	*P*_SNP_^a^_SZ_^c^	*P*_PGC_^d^
rs10497316	2	167,121,310	T	*XIRP2* ^e^	0.020 (0.0039)	3.8 × 10^−7^	0.98
rs17076524	6	146,985,464	T	*STXBP5-AS1*	0.018 (0.0036)	9.6 × 10^−7^	0.050
rs764113	7	109,040,240	A	*AC004014.3* (88 kb)	−0.012 (0.0026)	1.8 × 10^−6^	0.0043
rs12540954	7	109,047,522	T	*AC004014.3* (95 kb)	−0.012 (0.0026)	1.9 × 10^−6^	0.0050
rs10953591	7	109,048,681	C	*AC004014.3* (96 kb)	−0.012 (0.0026)	2.7 × 10^−6^	0.0048

^a^Based on human reference assembly GRCh38.p7

^b^Minor allele

^c^For the P-values presented here, FDR (Benjamini-Hochberg method) = 0.18

^d^GWAS *P*-values for SZ status as reported by the SZ Working Group of the Psychiatric Genomics Consortium (PGC), involving up to 36,989 SZ cases and 113,075 controls^8^

^e^In a recent gene-based association study of rare loss-of-function variants conducted by UK10K Consortium on whole-exome sequences of 4264 SZ cases, 9343 controls, and 1077 trios, the gene *XIRP2* yielded the strongest association with SZ status (*P* = 3.5 × 10^−5^)^37^

## Discussion

With the rapid expansion of transcriptome sequencing over the past decade, researchers have increasingly focused on the expression of genes relative to each other (i.e., co-expression) as a means of assigning putative functions to genes and ncRNAs based on the annotations of wider co-expression networks (so-called “guilt-by-association”), as well as gaining insight into potential regulatory relationships that underlie biological processes. Such network-based approaches reduce the inherent complexity and dimensionality of genome-wide expression data, as most genes are weakly correlated with other genes and thus disregarded, depending on chosen thresholds. Moreover, co-expression analyses have been used to identify novel risk genes for various human diseases, including psychiatric disorders, as coordinated gene expression is critical for brain development and function. This includes SZ, for which studies of postmortem brain specimens and peripheral blood have found dysregulated gene networks, including modules enriched for genes involved in synaptic transmission^38^, immune function^39,40^, oxidative stress and mitochondria^41,42^, and neurogenesis and neuron differentiation^42,43^.

In our network analysis of the genome-wide effects of DA perturbation on gene expression in LCLs, we examined the total sample, comprising both SZ cases and controls. Prior studies of the brain transcriptome have found gene co-expression patterns to be organized into distinct cellular and functional categories^44^, with significant overlap observed in gene membership of case- and control-only modules, suggesting that module composition and gene-gene connectivity per se are not likely to be key determinants in the pathogenesis of SZ^42^, although this does not preclude the possibility of certain regulatory relationships between smaller numbers of genes. Thus, our aim was to identify changes in co-expression levels within our networks that are associated with SZ status, to which one module, related to ribosomal translation, has revealed a marked increase in disease risk upon DA-stimulation in our sample.

Based on WGCNA, we identified six co-expression modules under baseline conditions that are preserved under DA-stimulation, both in terms of gene-gene network connectivities and significant overlap in gene membership with corresponding modules generated from the DA-stimulated data. When tested for their association with SZ status, the co-expression profiles (i.e., eigengenes) of the green baseline module (*n* = 127 genes) show the strongest effect, which is reflected by its median FDR of 1.1 × 10^−9^ for the genes within the module when analyzed individually in prior gene-based association results^16^. This module has a significant enrichment of genes related to inflammatory response, in particular TNF signaling and NF-kappa B signaling—cytokine pathways that are involved in systemic inflammation, apoptosis, immune response, and synaptic plasticity, with substantial evidence implicating them in the pathophysiology of SZ^45–48^. Under DA-stimulation, the corresponding module (*n* = 62 genes) also produced the most significant association with SZ, with the same pattern of primary enrichment for genes involved in inflammation and NF-kappa B and TNF signaling. These results are consistent with our previous studies on these expression data^16,20,22^, which found immune-related genes enriched among both baseline and DA-stimulated transcripts that are differentially expressed by affection status, as well as transcriptome studies by other groups^49^, thus further supporting the immune and cytokine hypothesis for SZ.

Overall, five of the seven baseline modules are strongly associated with SZ (*P* < 10^−5^), both for the baseline and DA-stimulated data, with the co-expression profiles of the unassigned genes also exhibiting a highly significant association (*P* = 4.3 × 10^−11^). This is consistent with our gene-based results, which showed a large number of SZ-associated gene expressions under both conditions (31 and 21%, respectively; FDR < 0.05). As previously discussed^16^, we did not find any evidence that these results are a consequence of any technical artifacts, and thus assume that they reflect real biology in our well-controlled LCL study. Intriguingly, this may reflect the recently proposed “omnigenic” model^50^, where most, if not all, genes outside core disease-related pathways indirectly contribute to disease risk, especially for transcriptomics and regulatory networks that involve higher-order, interconnected structures.

### DA-stimulated effects related to translational machinery

Of the six co-expression modules that correspond across the two conditions, only the brown module (*n* = 194 genes at baseline) shows stronger evidence of association with SZ after DA perturbation, with the estimated effect changing from −1.12 ± 0.96 (*P* = 0.023) to a highly significant −1.95 ± 0.96 (*P* = 7.8 × 10^−5^), with the latter representing a substantial 190-fold increase in model likelihood. Moreover, eight of the baseline module genes (4.1%) are in proximity to the 108 significant GWAS loci reported by the PGC for SZ, the highest proportion observed among our modules (empirical *P* = 0.079), seven of which are downregulated and exhibit more significant associations with SZ after DA-stimulation (top FDR = 1.7 × 10^−4^): *MRPS21*, *NDUFB3*, *NFATC3*, *RBX1*, *RPL13A*, *RPS11*, *SRP14*, and *ZFAS1*. Despite cellular stress and transcriptome-wide changes caused by the DA perturbation of the LCLs (~91% of genes responsive to DA at FDR < 0.05)^16^, gene-gene connectivity patterns in the brown module are strongly preserved in the DA-stimulated expression data (see Supplementary Table 1).

For the brown module under both conditions, but especially DA-stimulation, highly significant enrichments were observed for genes involved in signal recognition particle (SRP)-dependent targeting for endoplasmic reticulum (ER) translation (~134-fold; FDR = 2.7 × 10^−161^) and ribosomal translation (~43-fold; FDR = 1.1 × 10^−125^), accounting for 67.6 and 69.4% of the module genes, respectively. Coupled with the increased association with SZ under DA conditions, these results for the brown module are compelling, as ribosomal proteins and the broader translational machinery have been implicated in neurodevelopment and SZ in a number of recent papers. A gene knockdown study in the rat forebrain found neuronal maturation to be associated with a considerable expansion of ribosomal proteins, with translational insufficiency impairing dendritic growth and neuronal connectivity^51^. In another study involving human stem cell-derived neural progenitor cells from SZ patients and controls^52^, increased levels of global protein synthesis and translational machinery, including ribosomal proteins, were observed in the SZ cells. In contrast, for human olfactory neurosphere-derived cells, discovery-based proteomics and functional analyses revealed significant *reductions* in particular ribosomal proteins among SZ patients, including total ribosomal signal intensity^53^. And, perhaps most intriguingly, Zhou *et al*^54^. reveal that the interactome of *ZNF804A*, a SZ risk gene robustly replicated in different populations^55–58^, is highly represented by ribosomal and mitochondrial proteins, with ZNF804A modulating translational efficiency. Moreover, the ribosomal protein RPSA interacts with ZNF804A, and rescues neuronal migration and translational defects caused by knockdown of the *ZNF804A* homolog in mice, linking the SZ risk gene to neurodevelopment and translational control. Although *ZNF804A* was not assigned to our brown modules, *RPSA* was for both baseline and DA-stimulated data, and interestingly, the DA-stimulated expression levels of *RPSA* show a significant negative correlation with SZ (FDR = 3.9 × 10^−6^), with its differential expression between the two conditions (i.e., DA response) yielding the top association result among the 110 overlapping module genes (FDR = 0.027)^16^.

### Links to mitochondrial function?

Although we were unable to identify genome-wide significant QTLs for the brown module under DA-stimulation, the top results are nonetheless of interest. In particular, *FASTKD2* harbors a linkage disequilibrium block of strongly associated SNPs (minimum *P* = 2.8 × 10^−6^), with the gene playing a critical role in the biogenesis of mitochondrial ribosomes and translation^34,35^. The encoded RNA-binding protein modulates apoptosis, and is involved in the assembly of mitochondrial RNA granules, often induced under conditions of cellular stress, with abnormal accumulation of such granules linked to some neurodegenerative disorders^59,60^. Interestingly, the top enrichment result for the brown module, SRP-dependent targeting for ER translation, serves as a mechanism for mRNA to escape stress granule sequestration through localization to the ER^61^, which has obvious implications for neuronal cell survival. Furthermore, a SNP within *FASTKD2*, rs7594645, has been previously linked to memory performance in older adults, with carriers exhibiting neuroprotective effects, including increased hippocampal volume and gray matter density, and decreased cerebrospinal fluid levels of apoptotic mediators, all of which are features of Alzheimer’s disease^36^. And in a pair of interactome studies based on affinity capture-mass spectrometry^62,63^, *FASTKD2* was identified as a protein-protein interaction partner of *NGRN*, a SZ risk gene with downstream effects on ZNF804A in the aforementioned work by Zhou *et al*.^54^, as part of a larger oxidative phosphorylation (OXPHOS) network regulating mitochondrial translation.

For SNP*SZ interactions on DA-stimulated co-expression, rs10497316 in *XIRP2* was our top finding for the brown module (*P* = 3.8 × 10^−7^), reflecting a marked difference in the magnitude and direction of its main effect among SZ cases (*β* = 0.013 ± 0.0030; *P* = 6.77 × 10^−6^) versus controls (*β* = −0.0063 ± 0.0050; *P* = 0.014). The gene encodes a protein that protects actin filaments from depolymerization and is involved in neuronal integrity. In a large whole-exome sequencing study by the UK10K Consortium^37^, a disproportionately high number of LoF mutations was reported for *XIRP2* among SZ cases, corresponding to its top gene-based association for this class of variants (*P* = 3.5 × 10^−5^). Moreover, *XIRP* is a known target of MEF2A^64,65^, a DNA-binding transcription factor that mediates neuronal differentiation and survival, as it is cleaved by mitochondrial apoptotic caspases during excitotoxic neuronal stress^66^, and *XIRP2* has previously shown decreased expression in brain samples of PD patients^67^. For the other top interaction effects listed in Table 5, the SNPs all show nominal association with SZ in the PGC data (*P* < 0.05), and are positioned within or closest to lncRNAs, an abundant class of RNA molecules that regulate gene expression and are believed to play a critical role in neuronal development^68^, with prior co-expression network analyses implicating them in early onset SZ^69^.

Taken together, these SNP association results for the brown module suggest potential links with mitochondrial processes and cell survival/apoptosis, in particular neurodegeneration, as impaired ribosome production has been documented in neurodegenerative diseases^70–72^. This is borne out in our enrichment analyses for the KEGG lists in the baseline module (Fig. 1c; see Supplementary Table 6), as the next most enriched pathways after ribosomes are genes related to: OXPHOS in mitochondria (FDR = 2.4 × 10^−15^); Huntington’s disease (FDR = 9.0 × 10^−14^); Parkinson’s disease (FDR = 1.2 × 10^−12^); and Alzheimer’s disease (FDR = 3.9 × 10^−12^). For the DA-stimulated module, however, all four of these failed to show significant enrichment (minimum FDR = 0.20), stemming from the non-inclusion of 21 genes belonging to these pathways, 16 of which are shared. Taking a closer look at this splinter group, we found the association of its eigengenes with SZ status to change in a manner similar to the larger brown module (ΔAIC = −6.6), going from a non-significant association at baseline (*β* = −0.70 ± 0.94; *P* = 0.16) to a significant one (*β* = −1.44 ± 0.94; *P* = 3.50 × 10^−3^), with DA-stimulation weakening the co-expression between genes from this subgroup and the rest of the brown module, as evidenced by pairwise correlations in their eigengene scores (baseline: *r* = 0.81 ± 0.018; DA-stimulation: *r* = 0.69 ± 0.029).

Mitochondrial dysfunction characterizes various neurodegenerative diseases, reflected by the gene enrichments of the aforementioned KEGG pathways. Mitochondria play key roles in ATP generation, reactive oxygen species formation, and apoptosis, with neurons particularly dependent on mitochondria because of their high energy demands, and thus susceptible to cellular damage due to oxidative stress^73^. What is more, impaired mitochondrial function and variants in related genes have been repeatedly implicated in SZ^74–76^, with prior co-expression network analyses of SZ reporting similar modular enrichments^39,41,69^. SZ cases have been shown to exhibit abnormal mitochondrial respiration^77^, increased oxidative stress^78^, and altered gene expression of mitochondrialcomplexes^79^. Remarkably, in a recent study of pyramidal neurons from the DLPFC of postmortem SZ subjects, significant transcriptome alterations were identified in molecular pathways related to mitochondrial energy production *and* the regulation of protein translation, which corroborates our main findings^80^. These alterations of nuclear-encoded genes involved in energy production are consistent with the poor activation of DLPFC circuitry during increased cognitive demands that has been long observed in SZ^81^. Moreover, subjects responsive to antipsychotic treatment were identified in another study as having approximately a 40% decrease in the number of mitochondria per synapse in dorsal striatum at postmortem, with treatment-resistant cases having normal levels^82^, thus suggesting that there may be a biological distinction between treatment response and treatment resistance in SZ, for which DA may represent a key trigger. DA has been shown to have non-receptor-mediated effects on mitochondrial function, along with antipsychotics, as it inhibits Complex I activity and ATP production of the respiratory chain through its uptake into the organelle^83-86^. This potential link between DA and mitochondrial dysfunction in SZ risk is compelling and, given our findings, warrants further investigation.

## Conclusion

The results of our study identify a co-expression network that exhibits increased risk for SZ upon DA-stimulation of LCLs, highly enriched for ribosomal proteins and translational machinery. This suggests that relevant but cryptic pathological mechanisms underlying SZ can become detectable by functional perturbation, which makes replication of these findings more involved given our unique study design. Moreover, the use of LCLs as a cellular model versus brain presents some clear limitations for our study of a presumably brain-related disorder, as expression of some genes in LCLs will substantially differ from that in brain, although most expression signatures are shared between different tissues^87–91^. However, LCLs also have distinct advantages, as they are derived from the most accessible tissue, yielding sizeable samples, and allow for experimental manipulations such as DA perturbation, as we have discussed previously in greater detail^16^. Nonetheless, our findings provide key insights into the long-standing dopaminergic hypothesis for SZ based on gene co-expression changes, revealing non-receptor-mediated DA disturbances in translational machinery and mitochondrial function, including genes involved in neurodegeneration, with potential treatment targets of pre-synaptic dopaminergic features commonly observed in the disorder.

## Electronic supplementary material

Supplementary Material
