# Supplementary material for: Dopamine perturbation of gene co-expression networks reveals differential response in schizophrenia for translational machinery
Source: Transl Psychiatry. 2018 Dec 13;8:278. doi: 10.1038/s41398-018-0325-1 (PMC6293320; doi:10.1038/s41398-018-0325-1)

**SUPPL. TABLE 1. Preservation Statistics for Baseline Modules**

| **Baseline Module** | **Correlation *kIM**** | **Correlation *kME*^‡^** | **Median Rank^φ^** |
| --- | --- | --- | --- |
| Black | 0.88 | 0.98 | 5 |
| Blue | 0.94 | 0.96 | 6 |
| Brown | 0.99 | 0.99 | 1 |
| Green | 0.89 | 0.82 | 2 |
| Green-Yellow | 0.78 | 0.90 | 4 |
| Magenta | 0.90 | 0.94 | 9 |
| Purple | 0.81 | 0.64 | 4 |

*Correlation of intramodular connectivities (*kIM*), which are the summations of adjacency matrix values, or connection strengths, of individual module genes with other module genes.

^‡^Correlation of eigengene-based connectivities (*kME*), representing the correlation of gene expression levels with MEs of a given module.

^φ^Median rank of preservation statistics outputted by the R command “modulePreservation” in the WGCNA package.

**SUPPL. TABLE 2. Top Hub Genes for WGCNA Modules**

|  | **Baseline** | | **DA-Stimulated** | |
| --- | --- | --- | --- | --- |
| **Module Color** | **Hub Gene** | ***kIM**** | **Hub Gene** | ***kIM**** |
| Black | *HNF1B* | 33.3 | *HNF1B* | 9.8 |
| Blue | *ERLEC1* | 22.7 | *MANF* | 4.7 |
| Brown | *RPL11* | 20.5 | *RPL11* | 11.4 |
| Green | *NFKB2* | 8.9 | *NFKB2* | 3.9 |
| Green-Yellow | *ATAD2* | 8.5 | *CCNA2* | 5.8 |
| Magenta | *CCT6A* | 13.4 | *DKC1* | 2.9 |
| Purple | *MUC4* | 4.8 | – | – |

*The top intramodular connectivities (*kIM*) of the modules, corresponding to the listed hub gene.

**SUPPL. TABLE 3. Top Functional Annotation Clusters for WGCNA Modules**

|  | **Baseline** | | **DA-Stimulated** | |
| --- | --- | --- | --- | --- |
| **Module Color** | **Annotation Term(s)** | **Enrichment Score*** | **Annotation Term(s)** | **Enrichment Score*** |
| Black | Antiviral defense; immunity | 13.8 | GTPase activity | 1.9 |
| Blue | ER-Golgi transport | 22.1 | Endoplasmic reticulum | 12.8 |
| Brown | Ribosomal translation | 88.9 | Ribosomal translation | 116.6 |
| Green | TNF and NFkB signaling | 2.8 | NFkB signaling | 4.5 |
| Green-Yellow | Mitosis | 44.2 | Mitosis | 57.9 |
| Magenta | Mitochondria | 12.0 | Chaperone | 9.5 |
| Purple | Epidermal growth factor-like domain | 1.3 | – | – |

*Functional annotation clustering was performed in DAVID v. 6.8. Enrichment scores represent the geometric means of the Fisher’s Exact P-values for individual gene sets tested in the primary analyses.

**SUPPL. TABLE 4. Associations between DA-Stimulated Co-Expression Modules and SZ Risk**

|  |  |  | **Baseline Data*** | | |  |
| --- | --- | --- | --- | --- | --- | --- |
| **DA-Stimulated Module** | **Beta (SE)** | ***P*-Value** | **Beta (SE)** | ***P*-Value** | **ΔAIC^Ψ^** | **% PGC Loci^φ^** |
| Black | 2.70 (0.49) | 4.1 × 10^-8^ | 2.94 (0.49) | 2.0 × 10^-9^ | -5.9 | 2.0 |
| Blue | -2.67 (0.49) | 5.9 × 10^-8^ | -3.06 (0.49) | 4.8 × 10^-10^ | -9.4 | 0 |
| Brown | -1.92 (0.49) | 1.0 × 10^-4^ | -1.17 (0.49) | 0.018 | 9.6 | 4.5 |
| Green | 3.88 (0.48) | 2.0 × 10^-15^ | 4.35 (0.49) | < 2.0 × 10^-16^ | -16.8 | 0 |
| Green-Yellow | -0.71 (0.49) | 0.15 | -0.76 (0.49) | 0.13 | -0.3 | 4.7 |
| Magenta | 0.83 (0.49) | 0.093 | 2.22 (0.49) | 6.6 × 10^-6^ | -17.5 | 3.1 |
| Grey (Unassigned) | 2.64 (0.49) | 7.8 × 10^-8^ | 3.45 (0.49) | 2.1 × 10^-12^ | -20.6 | 2.5 |

*For the DA-stimulated WGCNA modules, the eigengenes were recalculated based on the DA-stimulated gene expression data, which were then tested for association with SZ status.

^Ψ^Difference in the Akaike Information Criteria (AIC) values for baseline and DA-stimulated regression models.

^φ^Percentage of SZ risk genes in a given module. This is based on the findings of the PGC GWAS on SZ, in which 108 SNPs and indels were identified as genome-wide significant, which were assigned to genes and ncRNAs using a 250K bp window around the loci. The percentage of unassigned genes that are PGC risk genes is 2.5%.

**SUPPL. TABLE 5. Top-5 Genome-Wide SNP × SZ Interactions for Brown Module Eigengenes for Baseline Data**

| **SNP** | **Chrom.** | **Position (bp)*** | **MA^§^** | **Gene/ncRNA** | **Beta (SE)** | **P_SNP*SZ_^‡^** | **P_PGC_^φ^** |
| --- | --- | --- | --- | --- | --- | --- | --- |
| rs10926354 | 1 | 240,779,800 | T | *RGS7* | -0.015 (0.0029) | 2.7 × 10^-7^ | 0.093 |
| rs12097299 | 1 | 240,779,832 | C | *RGS7* | -0.015 (0.0029) | 5.9 × 10^-7^ | 0.093 |
| rs2396541 | 7 | 112,465,814 | T | *IFRD1* | 0.017 (0.0035) | 1.0 × 10^-6^ | 0.049 |
| rs11135806 | 8 | 24,520,523 | G | *ADAM7* | 0.016 (0.0033) | 2.0 × 10^-6^ | 0.60 |
| rs4872244 | 8 | 24,527,015 | G | *ADAM7* | 0.016 (0.0033) | 2.2 × 10^-6^ | 0.59 |

*Based on human reference assembly GRCh38.p7.

^§^Minor allele.

^‡^ For the P-values presented here, the FDR scores (Benjamini-Hochberg method) are the following: 0.18, 0.20, 0.23, 0.25, and 0.25.

^φ^GWAS P-values for SZ status as reported by the Schizophrenia Working Group of the Psychiatric Genomics Consortium (PGC), involving up to 36,989 SZ cases and 113,075 controls.^8^

**SUPPL. TABLE 6. Top-5 KEGG Pathway Enrichment Results for the Brown Module**

| **Baseline** | |  | **DA-Stimulated** | |
| --- | --- | --- | --- | --- |
| **Top-5 KEGG Pathways*** | **FDR** |  | **Top-5 KEGG Pathways*** | **FDR** |
| hsa03010: Ribosome | 2.3 × 10^-110^ |  | hsa03010: Ribosome | 1.1 × 10^-125^ |
| hsa00190: Oxidative phosphorylation | 2.4 × 10^-15^ |  | hsa00190: Oxidative phosphorylation | 0.20 |
| hsa05016: Huntington's disease | 9.0 × 10^-14^ |  | hsa05012: Parkinson's disease | 0.25 |
| hsa05012: Parkinson's disease | 1.2 × 10^-12^ |  | hsa05016: Huntington's disease | 0.26 |
| hsa05010: Alzheimer's disease | 3.9 × 10^-12^ |  | hsa05010: Alzheimer's disease | 0.41 |

*KEGG pathways tested for gene enrichment in DAVID v. 6.8.

**SUPPL. FIGURE 1.** Boxplots of the variance explained (%) in log2 transformed RPKM expression levels under baseline condition (*n* = 21,043 genes) of the following covariates: sex, age, cell counts and ATP levels at cell harvest, genotypic ancestry principal components (PCs 1-5), and sequencing batch. The analysis was performed using the R program variancePartition v. 3.7.

**
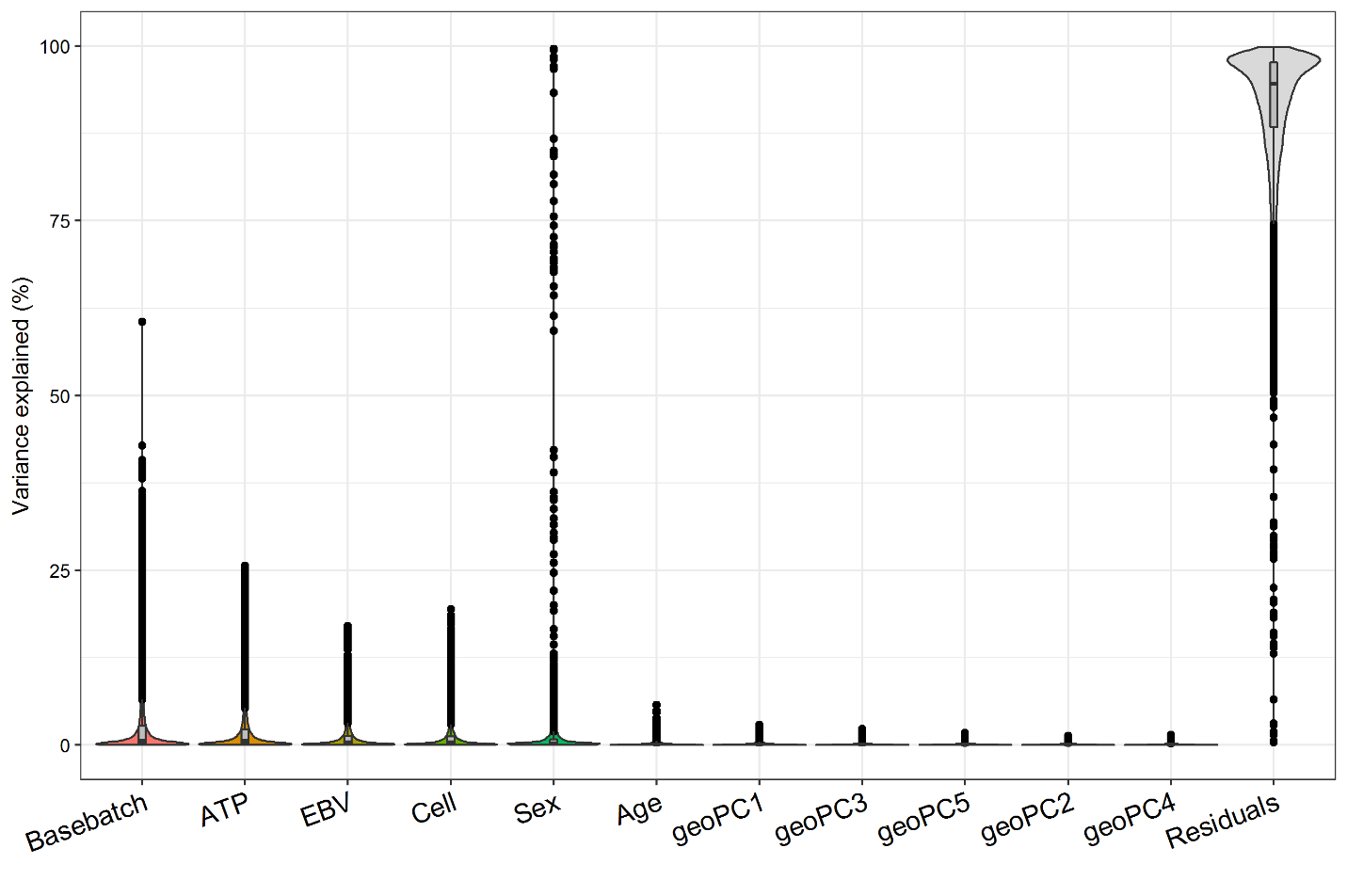
**

**SUPPL. FIGURE 2.** Boxplots of the variance explained (%) in log2 transformed RPKM expression levels under DA-stimulation (*n* = 21,043 genes) of the following covariates: sex, age, cell counts and ATP levels at cell harvest, genotypic ancestry principal components (PCs 1-5), and sequencing batch. The analysis was performed using the R program variancePartition v. 3.7.

**
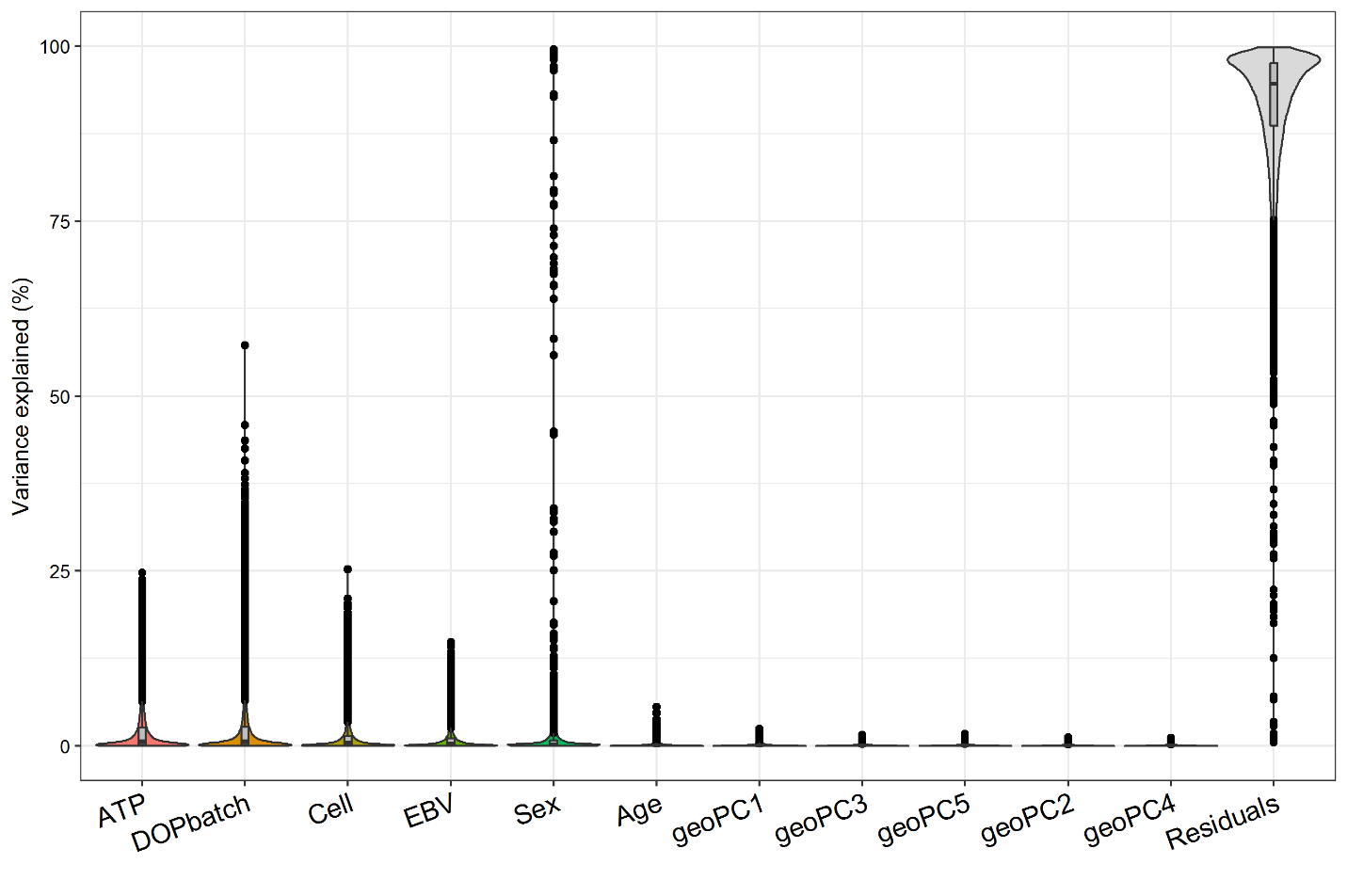
**

**SUPPL. FIGURE 3.** UPGMA dendrogram of TOM dissimilarity scores for baseline gene expression data (*n* = 21,043). Via the WGCNA command “cutreeDynamic”, 13 co-expression modules were identified in the dendrogram as shown (“grey” are unassigned genes), reduced to seven after merging at DistME < 0.25.


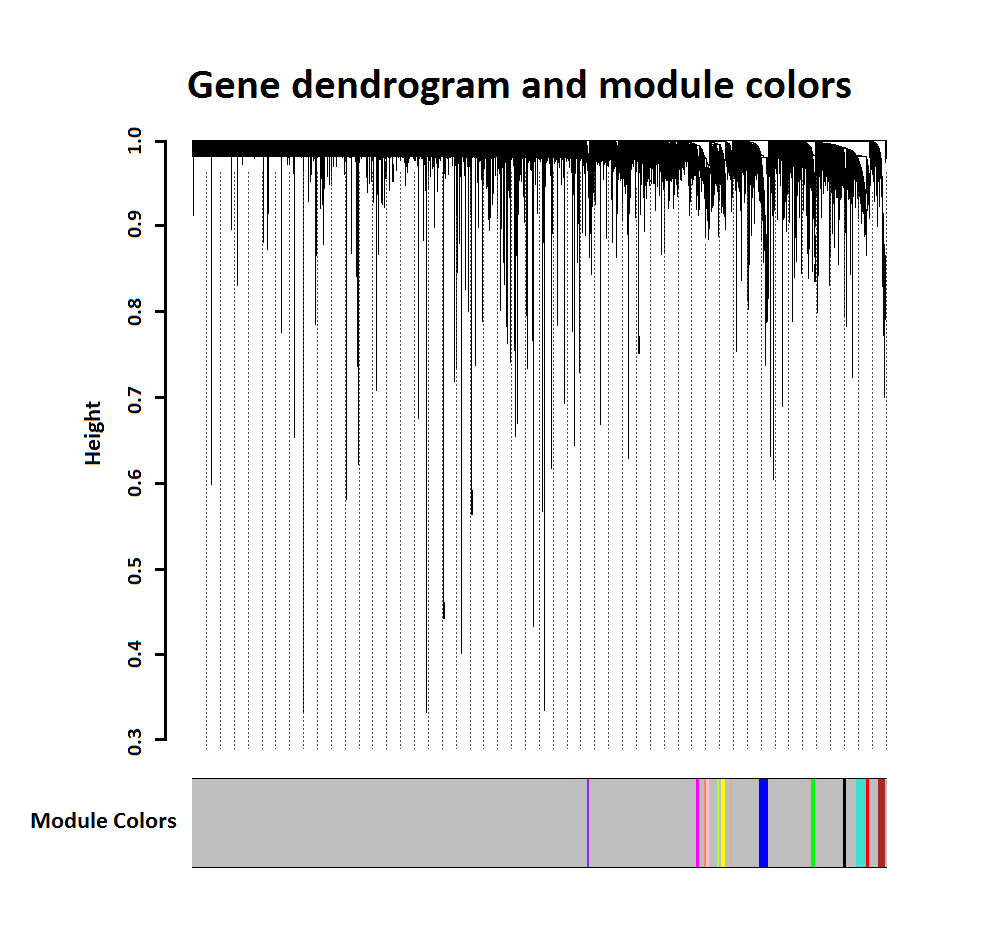


**SUPPL. FIGURE 4.** UPGMA dendrogram of TOM dissimilarity scores for DA-stimulated gene expression data (*n* = 21,043). Via the WGCNA command “cutreeDynamic”, seven co-expression modules were identified in the dendrogram as shown (“grey” are unassigned genes), reduced to six after merging at DistME < 0.25.


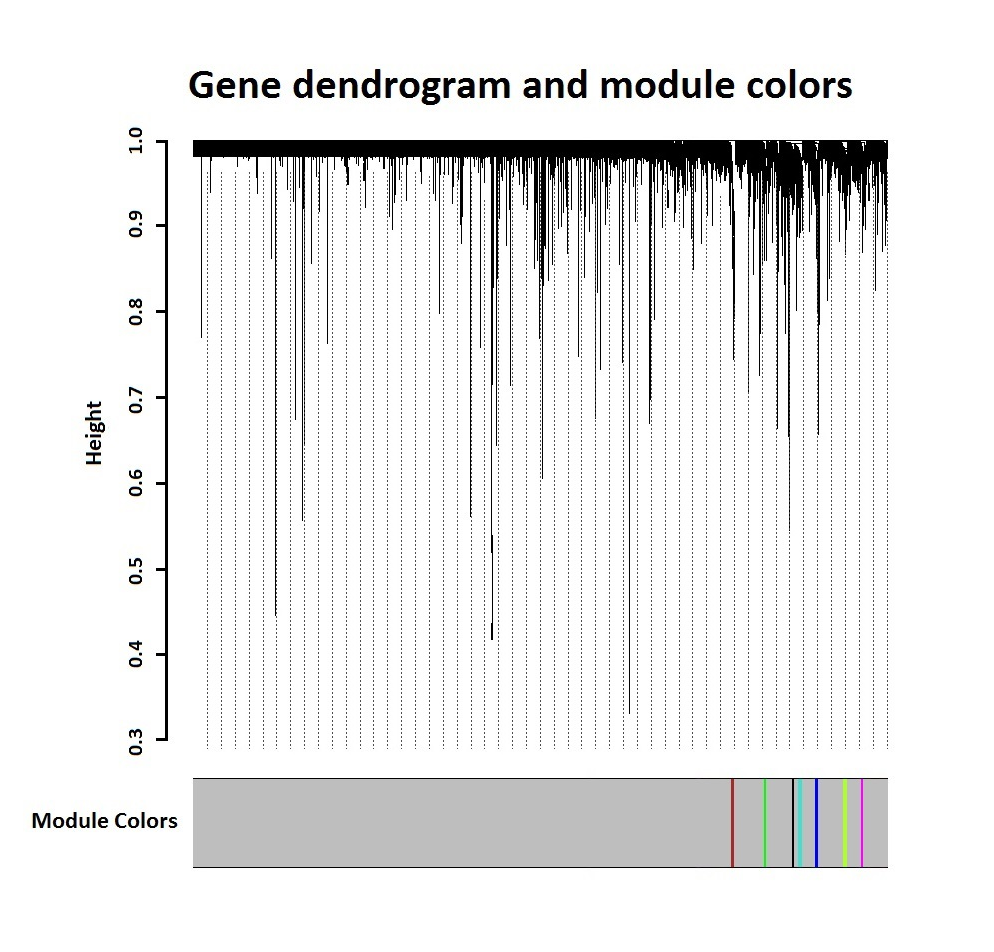

Supplement: Supplementary file 1 — Supplementary Material [file 41398_2018_325_MOESM1_ESM.docx]
